# Supplementary material for: Dynalign II: common secondary structure prediction for RNA homologs with domain insertions
Source: Nucleic Acids Res. 2014 Nov 21;42(22):13939–48. doi: 10.1093/nar/gku1172 (PMC4267632; doi:10.1093/nar/gku1172)
Supplement: SUPPLEMENTARY DATA [file supp_gku1172_nar-02021-z-2014-File012.zip › manual/RNA_class/html/files.html]

RNAstructure Classes: File Index

- Main Page
- Classes
- Files

- File List
- File Members

# File List

Here is a list of all files with brief descriptions:

|  |  |
| --- | --- |
| F:/RNAstructure-co-from-repository/RNAstructure/RNA\_class/Dynalign\_class.cpp |  |
| F:/RNAstructure-co-from-repository/RNAstructure/RNA\_class/Dynalign\_object.cpp |  |
| F:/RNAstructure-co-from-repository/RNAstructure/RNA\_class/Dynalign\_object.h |  |
| F:/RNAstructure-co-from-repository/RNAstructure/RNA\_class/DynalignSMP\_class.cpp |  |
| F:/RNAstructure-co-from-repository/RNAstructure/RNA\_class/HybridRNA.cpp |  |
| F:/RNAstructure-co-from-repository/RNAstructure/RNA\_class/HybridRNA.h |  |
| F:/RNAstructure-co-from-repository/RNAstructure/RNA\_class/HybridRNA\_class.cpp |  |
| F:/RNAstructure-co-from-repository/RNAstructure/RNA\_class/Multifind\_object.cpp |  |
| F:/RNAstructure-co-from-repository/RNAstructure/RNA\_class/Multifind\_object.h |  |
| F:/RNAstructure-co-from-repository/RNAstructure/RNA\_class/Multilign\_object.cpp |  |
| F:/RNAstructure-co-from-repository/RNAstructure/RNA\_class/Multilign\_object.h |  |
| F:/RNAstructure-co-from-repository/RNAstructure/RNA\_class/OligoWalk\_class.cpp |  |
| F:/RNAstructure-co-from-repository/RNAstructure/RNA\_class/Oligowalk\_object.cpp |  |
| F:/RNAstructure-co-from-repository/RNAstructure/RNA\_class/Oligowalk\_object.h |  |
| F:/RNAstructure-co-from-repository/RNAstructure/RNA\_class/RNA.cpp |  |
| F:/RNAstructure-co-from-repository/RNAstructure/RNA\_class/RNA.h |  |
| F:/RNAstructure-co-from-repository/RNAstructure/RNA\_class/RNA\_class.cpp |  |
| F:/RNAstructure-co-from-repository/RNAstructure/RNA\_class/thermodynamics.cpp |  |
| F:/RNAstructure-co-from-repository/RNAstructure/RNA\_class/thermodynamics.h |  |
| F:/RNAstructure-co-from-repository/RNAstructure/RNA\_class/TwoRNA.cpp |  |
| F:/RNAstructure-co-from-repository/RNAstructure/RNA\_class/TwoRNA.h |  |

---

Generated on Tue Jul 30 10:52:29 2013 for RNAstructure Classes by 
 1.5.7.1
